# Intranasal Corticosteroids and Oral Montelukast for Paediatric Obstructive Sleep Apnoea: A Systematic Review

**DOI:** 10.3390/pharmaceutics17050588

**Published:** 2025-04-30

**Authors:** Marco Zaffanello, Angelo Pietrobelli, Luana Nosetti, Franco Antoniazzi, Rossella Frassoldati, Giorgio Piacentini

**Affiliations:** 1 Department of Surgical Sciences, Dentistry, Gynecology and Pediatrics, University of Verona, 37126 Verona, Italy; angelo.pietrobelli@univr.it (A.P.); franco.antoniazzi@univr.it (F.A.); giorgio.piacentini@univr.it (G.P.); 2 Pediatric Department, Department of Medicine and Technological Innovation, Insubria University, F del Ponte Hospital, 21100 Varese, Italy; luana.nosetti@uninsubria.it; 3 Department of Pediatrics, Women’s & Child’s University Hospital, 37126 Verona, Italy; rossella.frassoldati@aovr.veneto.it

**Keywords:** adenoid hypertrophy, children, corticosteroid, infant, montelukast, obstructive sleep apnoea, sleep-disordered breathing

## Abstract

**Background/Objectives:** Paediatric Obstructive Sleep Apnoea (OSA) is characterised by recurrent episodes of upper airway obstruction during sleep, manifesting as snoring, intermittent oxygen desaturation, and frequent nocturnal awakenings. Standard treatments include surgical interventions, pharmacological therapies, intranasal corticosteroids, and oral montelukast. However, significant variability exists across studies regarding dosage and outcome assessment. This literature review systematically evaluated clinical evidence regarding the efficacy and safety of intranasal corticosteroids and oral montelukast for treating sleep-disordered breathing and its primary underlying condition, adenoid hypertrophy, in otherwise healthy children. **Methods:** The MEDLINE (PubMed), Scopus, and Web of Science databases were systematically searched up to 13 February 2025, using tailored search terms combining keywords and synonyms related to paediatric OSA, adenoidal hypertrophy, corticosteroids, montelukast, and randomised controlled trials. Owing to variability in outcome measures, Fisher’s method for *p*-value combination was employed to enable a comprehensive comparison of drug effects. **Results:** Available evidence shows that intranasal corticosteroids (mometasone, beclometasone, budesonide, fluticasone, and flunisolide), either as monotherapy or in combination with other agents, consistently lead to clinical and instrumental improvements in adenoid hypertrophy and related respiratory symptoms, with a generally favourable safety profile. Combining montelukast with intranasal corticosteroids appears to offer superior benefits compared with monotherapy. Nevertheless, the reviewed studies varied widely in dosage, treatment duration, design, and sample size. The reported side effects are mostly mild; however, long-term studies are lacking to establish the complete safety of these treatments in children. **Conclusions:** Intranasal corticosteroids and oral montelukast effectively and safely manage adenoid hypertrophy and mild-to-moderate OSA symptoms in children. Nonetheless, the heterogeneity of study designs necessitates larger prospective trials with standardised protocols and more extended follow-up periods to draw more robust conclusions. Future studies should aim to stratify treatment outcomes based on OSA severity and duration to tailor therapeutic approaches better.

## 1. Introduction

Obstructive Sleep Apnoea (OSA) is a disorder characterised by recurrent episodes of upper airway obstruction during sleep, associated with snoring, noisy breathing, intermittent desaturation, and nocturnal awakenings [1]. OSA affects approximately 3% of preschool- and school-aged children [2]. The most common cause is adenotonsillar hypertrophy (ATH) [2]. Other risk factors include obesity, craniofacial abnormalities, and neuromuscular diseases, although they are less common [2]. Children with OSA may present with habitual snoring, restless sleep, mouth breathing, and attention and mood disorders more frequently [3].

The gold standard for diagnosis is overnight Polysomnography (PSG). However, owing to the limited availability of paediatric sleep laboratories, screening questionnaires have been developed to identify children at risk of OSA, such as the Paediatric Sleep Questionnaire (PSQ) and OSA-18 [4,5]. An anatomical evaluation of the upper airway is required to complete the diagnostic assessment. In particular, adenoid size is assessed through nasopharyngeal endoscopy and/or radiological imaging [6,7].

The treatment of paediatric OSA is selected based on the severity of the clinical polysomnographic profile and the underlying causes [8]. In moderate-to-severe forms, adenotonsillectomy is the first-line treatment for significant ATH [9]. Medical treatment is available in mild-to-moderate cases, or when surgery is contraindicated. Specifically, local and systemic anti-inflammatory therapies may have therapeutic roles in paediatric OSA [9,10]. Intranasal corticosteroids and leukotriene antagonists, such as oral montelukast, may improve symptoms and reduce polysomnographic severity indices [11].

Intranasal corticosteroids reduce nasal mucosal oedema and inflammatory mediator production (IL-6, IL-1β, and TNF-α) [12] and decrease adenoid volume [12]. Montelukast inhibits the cysLT1 receptor, reduces eosinophilic infiltration and oedema in lymphoid tissues [13], decreases the expression of COX-2 and 5-lipoxygenase, and reduces circulating levels of IL-8 and high-sensitivity CRP [14,15].

Literature from the past decade suggests that intranasal corticosteroids can improve symptoms related to adenoid hypertrophy (AH). A meta-analysis of eight RCTs demonstrated that mometasone significantly reduced nasal obstruction and the adenoid-to-choana ratio (A/C ratio) [16]. Another meta-analysis, which included five trials (mometasone furoate, fluticasone propionate, and budesonide), also conducted on adults, revealed a high risk of bias and considerable methodological heterogeneity [17].

Oral montelukast, evaluated in four RCTs involving children with mild to moderate OSA, significantly improved polysomnographic parameters [15]. However, a review of five trials, three on intranasal corticosteroids and two on montelukast, with the Apnoea–Hypopnea index (AHI) as the primary outcome, demonstrated insufficient evidence supporting corticosteroid efficacy and only short-term benefits for montelukast, without long-term safety data [9]. Another study involving children aged 1 to 16 years confirmed the short-term positive effects of intranasal corticosteroids and montelukast on desaturation indices and oxygen saturation [18].

The combined use of montelukast and intranasal corticosteroids, as well as montelukast monotherapy, also appears effective in the short-term management of paediatric OSA (reduction in AHI) [11] and in the treatment of AH [19]. In an analysis of 17 RCTs involving children aged 2–14 years with OSA, mometasone combined with montelukast, budesonide, and montelukast monotherapy showed superiority over placebo in improving AHI [20]. Similarly, combined treatment with mometasone and montelukast was more effective than mometasone alone in improving symptoms and adenoid-to-nasopharynx ratio (A/N ratio) [21].

A traditional meta-analysis approach may be complex or less informative because of the significant heterogeneity in the primary outcomes reported in previous studies. Fisher’s Combined Probability Test enables aggregating results based on *p*-values, avoiding methodological issues associated with data transformation into a single standard measure. Moreover, this method determines the overall significance of the association between the intervention and outcome [22,23].

## 2. Materials and Methods

### 2.1. Search Strategy and Study Selection

We searched three major databases—MEDLINE (PubMed), Scopus, and Web of Science—for English-language studies published up to 13 February 2025. MeSH terms and text words (and their combinations and truncated synonyms) were used. Customised search terms were adapted for each database, combining keywords and synonyms: (children OR pediatric) AND (obstructive sleep apnoea OR sleep-disordered breathing OR adenoidal hypertrophy) AND (corticosteroids OR montelukast) AND (randomised controlled study). The search terms used are presented in Appendix A.

Studies were eligible for inclusion if they were

(1) The paediatric population.
(2) Assessed the effects of intranasal corticosteroids and/or montelukast in treating Obstructive Sleep Apnoea (OSA), sleep disordered breathing (SDB), and adenoid hypertrophy (AH).
(3) Employed a randomised controlled design.
(4) Reported the clinical and/or instrumental outcomes.

We excluded studies that did not meet these criteria, non-English publications, animal studies, and studies involving surgical or non-pharmacological interventions. No restrictions were placed on geographical region, sex, or specific age ranges within the paediatric population. No particular outcome domains were predefined to allow the inclusion of all clinically relevant findings compatible with the scope of the review.

Duplicate articles were removed before screening the abstracts. Two authors (MZ and LN) reviewed the titles and abstracts independently, followed by full-text assessments. Discrepancies were resolved through discussion or adjudication by a third reviewer (AP). The reference lists of the included studies were screened for additional eligible articles. Ethical approval was not required because the study did not involve human participants. The systematic review followed the PRISMA guidelines [24].

### 2.2. Statistics

The AI tools (QUADAS-2 and QUADAS-C, ASReview, Abstrackr, RobotAnalyst, EPPI-Reviewer, and DistillerSR) are a promising innovation for systematic review practices [25,26]. During the revision process, Generative AI (ChatGPT 4 and 4o, Plus plan, OpenAI) [27,28] was used to explore appropriate statistical approaches for synthesising heterogeneous *p*-values derived from RCT.

To comprehensively compare the effect of the drug versus control across these studies, considering the variety of outcome measures and the predominant availability of *p*-values, we employed the probability combination method known as Fisher’s [22,23].

Using Fisher’s method, the statistic $X_{2k}^{2}=-2\sum_{i=1}^{k}\ln\left(p_{i}\right)$, where $p_{i}$ is the *p*-value from the *i*th test, and *k* is the total number of combined tests. The resulting variables *X^2^* approximately follow a chi-square distribution with 2*k* degrees of freedom, calculated as twice the number of studies included. By comparing this *X*^2^ value against critical values from the chi-square distribution with 2*k* degrees of freedom (as found in the chi-square distribution tables), an overall combined *p*-value was obtained [29].

The AI tool also assisted in designing a datasheet for applying Fisher’s method of *p*-value combinations. The detailed procedure for calculating the combined *p*-values using Fisher’s method in Microsoft^®^ Excel^®^ for Microsoft 365 MSO (Version 2503) is provided in Appendix B, Table A1.

The main *p*-values reported by each study were selected by identifying the value(s) representing the primary endpoint or the most directly comparable outcome across the trials. For each study, at least one *p*-value for the difference between the treatment and control groups was considered. This approach allowed for an overall estimation of treatment effectiveness, even when individual studies reported distinct *p*-values without sufficiently homogeneous data. The authors critically reviewed and approved all the analyses, decisions, and interpretations.

## 3. Results

**Figure 1:**
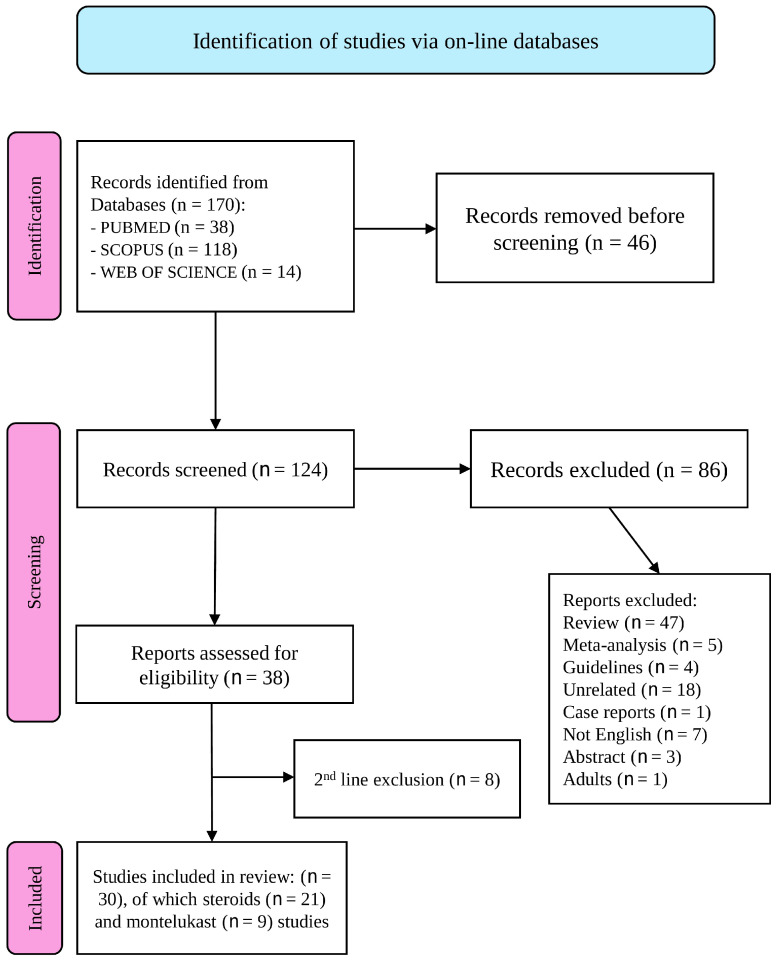
The PRISMA flow diagram [24] visually represents the study selection process and number of studies included at each stage. For more information, visit: http://www.prisma-statement.org/ (accessed on 16 February 2025).

In total, 170 articles were identified, of which 46 were duplicates. Consequently, 124 studies were included (Figure 1). After the selection process, 40 articles were included in the final analysis. After full-text analysis, an additional 10 studies were examined and excluded. Overall, 30 studies met the inclusion criteria.

Table 1 summarises the controlled clinical trials, including randomised [30,31,32,33], double-blind (placebo-controlled) [30,31,32], crossover [34], and multi-centre studies [31]. Some studies included larger sample sizes (*n* > 200) [31], whereas others enrolled fewer than 30 participants [34]. Inclusion criteria were clearly defined, targeting children with AH [30,34,35,36] associated with obstructive respiratory symptoms [31,32] and otitis media with effusion (OME) [35,36].

The exclusion criteria were recent steroid use [30,31,32,33,34,35,36], craniofacial anomalies or malformations [30,31,32,33,35,36], and allergic or immunodeficiency conditions [30,33,34,36].

All studies measured at least one objective parameter (e.g., adenoid volume reduction via endoscopy, radiography, A/C ratio), obstructive AHI—oAHI—on PSG (etc.), and subjective symptoms (snoring and mouth breathing). Many studies have employed validated tools such as the PedsQL or the Glasgow Children’s Benefit Inventory [31]. One study extensively analysed polysomnographic parameters (AHI and ODI) [32]. Studies with greater methodological robustness (multi-centre, large sample size, double-blind) and others that were smaller [34] or shorter in duration [36].

The dosage of mometasone furoate typically ranges from 50 to 100 µg per nostril per day (100–200 µg/day total), administered once or twice daily. Some protocols begin with two puffs per nostril per day [35], others with a single puff [30], occasionally followed by alternate-day maintenance or reduced dosing [30,35]. The treatment duration varied from 6 weeks [36] to 3–4 months [30], 6 months [35], or a one-year follow-up [33]. Some studies have reported reduced sample sizes and significant dropout rates [32]. Most comparisons were between mometasone furoate and placebo (saline solution) [30,31,32,33,34,35].

The most significant studies [30] showed decreased endoscopic or radiological obstruction (A/C ratio). One study did not find statistically significant differences in adenoid volume, but noted symptomatic improvement [34]. Most studies reported reduced severity scores for symptoms (apnoea, snoring, and nasal obstruction) in the mometasone group compared to controls [30,32,35,36]. One study documented a significant reduction in polysomnographic parameters (oAHI and ODI) [32]. In cases of OME, some studies demonstrated quicker radiological and clinical resolution/improvement in patients treated with mometasone [35,36]. Other studies reported decreased symptom severity scores and reduced perceived need for adenoidectomy [31]. Relevant adverse events were rare; one study noted mild epistaxis [30], but mometasone use was generally tolerated.

Considering the statistical analysis of *p*-values reported, due to the very small individual *p*-values (except for one study with *p* = 0.51) [33], the sum of log-transformed *p*-values was extremely low. The resulting χ^2^ statistic far exceeded the critical threshold of a χ^2^ distribution with 2k degrees of freedom. Notably, one study reporting a non-significant *p*-value for the ‘resolution of SDB symptoms’ (*p* = 0.51) still indicated significant differences in other outcomes, such as quality of life scores (*p* = 0.001) [33].

The studies presented in Table 2 [37,38,39] aimed to evaluate the effectiveness of nasal beclomethasone spray in reducing AH and associated obstructive symptoms such as nasal obstruction, snoring, and related issues (e.g., OME). All studies were randomised and double-blind, with some including a crossover study component.

Despite some differences in the study design (duration, dosage, and outcome measures), there is a general trend toward moderate methodological quality. Specifically, the enrolled sample sizes were relatively small, ranging from a minimum of 20 children [39] to a maximum of 60 children [38], with notable dropout rates during follow-up [39].

Regarding therapeutic protocols, all three studies used nasal beclomethasone (generally 200–400 µg/day) for periods ranging from 8 weeks [37] to 24 weeks [38]. One study began with a higher initial dose, followed by a maintenance phase at a reduced dose [38]. The follow-up durations varied from 8 weeks [37] to 24 weeks [39]. In one case, the authors followed the children for up to 100 weeks [38]. However, only a part of the protocol was double-blind and controlled.

Concerning efficacy, one study reported modest and sometimes non-significant reductions in adenoid size documented through instrumental examinations [37]. Another study demonstrated a 29% reduction in the adenoid area after 24 weeks of therapy [39], significantly improving obstructive symptoms.

In the study by Lepcha et al. [37], *p*-values ranged from 0.71 to 1.00 for most variables, with the lowest value of 0.07 for endoscopy; thus, a *p*-value of 0.07 was selected for the endoscopic endpoint. Criscuoli et al. [38] reported *p*-values of 0.71 (nasal obstruction), 0.30 (snoring), and 0.39 (nasal discharge), along with values greater than 0.05 for endoscopic and radiographic findings. Since all values exceeded 0.05, a representative *p*-value of 0.30 (snoring) was selected. Demain and Goetz [39] reported significantly lower *p*-values for the A/C ratio reduction (*p* = 0.0002 right, *p* = 0.0006 left) and around 0.05 for symptomatic scores. The smallest and most statistically significant *p*-value for adenoid reduction was 0.0002.

Although two studies reported non-statistically significant results for the primary endpoints [37,38], the pronounced effect observed by Demain and Goetz [39] (*p* = 0.0002) strongly influenced Fisher’s combined analysis, resulting in a highly significant global *p*-value (*p* < 0.001). Fisher’s combined *p*-value analysis suggested an overall benefit of beclometasone compared to controls (*p* < 0.001). However, discrepancies among studies (two showing no significant differences [37,38]), one demonstrating significance [39], and the variability of outcomes warrant caution in clinical interpretation.

The studies presented in Table 3 evaluate the effects of beclometasone on AH and symptoms related to SDB [40,41,42]. These studies exhibited good methodological quality: prospective, randomised, placebo-controlled, and double blind. Inclusion and exclusion criteria were clearly defined, with sufficiently large populations ranging from 60 [40] to 100 children [41]. However, not all studies have provided long-term follow-up [40,42].

Budesonide was administered at dosages between 64 µg [41] and 128 µg [40] per day via nasal spraying [41] or transnasal nebulisation. The treatment duration ranged from six weeks [40] to approximately 14 weeks [41]. One study included additional observation weeks to monitor potential recurrence [41]. In one case, a two-week double-blind phase (with nebulised budesonide or placebo) was followed by a twelve-week open-label phase using a nasal spray [41].

Clinically, the results confirmed significant improvement in SDB, including reduced nasal obstruction and snoring [40] and decreased AHI scores in mild-to-moderate OSA cases [42]. Positive impacts on the quality of life of patients and their families have also been reported [40]. Additionally, some studies have documented a reduction in the adenoid-to-nasopharynx ratio [42].

From a safety and tolerability perspective, the adverse events reported were mild and clinically insignificant. The study by Gudnadottir et al. included various outcome measures, such as OSA-18, sleep disturbances, caregiver concerns, and quality of life, reporting a *p*-value of 0.0014 for the OSA-18 score (considered the most comprehensive variable) [40].

Hong et al. reported *p*-values < 0.001 for the nasal obstruction index (NOI), snoring, and nasal discharge, while the adenoidectomy rate had a *p*-value of 0.002 [41]. Considering NOI as the primary endpoint, we assumed an indicative *p* value of 0.0005 (between 0.0001 and 0.001, representing *p* < 0.001). Kheirandish-Gozal and Gozal reported *p*-values of <0.0001 for oAHI, 0.001 for the arousal index (RAI), 0.004 for Nadir SpO_2_, and again <0.0001 for the adenoid/nasopharynx (N/P) ratio. Typically, oAHI is the primary endpoint in OSA studies; hence, a reference value of *p* = 0.0001 (equivalent to *p* < 0.0001) was selected for calculations [42]. For a χ^2^ distribution with 6 degrees of freedom, a value of approximately 46.76 corresponds to a *p*-value of <0.0001, favouring budesonide efficacy compared to placebo in the considered studies.

The three clinical trials presented in Table 4 investigate the treatment of AH and related symptoms of SDB using fluticasone [43,44,45]. Each of these studies follows an RCT methodology, but differs in design (open-label [43] vs. blinded [44]).

In the study by Esteitie et al., conducted on 24 children aged 2 to 12 years diagnosed with OSA (AHI ≥ 5/hour), nasal spray fluticasone furoate (55 µg per nostril, once daily) was administered for only two weeks compared to an untreated control group [43]. Although prospective, this study was not blinded, introducing potential observer bias. The primary outcome was evaluating an inflammatory marker (IL-6) in adenoid tissue. Children treated with steroids showed a significant reduction in IL-6 compared to untreated controls, while other mediators (IL-10, TGF-β, TNF, etc.) showed no relevant changes [43].

The study by Brouillette et al. is a triple-blind randomised trial involving 25 children with mild-to-moderate OSA [44]. Treatment consisted of nasal fluticasone at 200 µg/day for one week, reduced to 100 µg/day for the subsequent five weeks, compared to a placebo. Results showed a significant reduction in AHI and ODI in the steroid-treated group [44].

Demirhan et al. evaluated the efficacy of fluticasone propionate (400 µg/day) nasal drops for eight weeks compared to saline solution in 45 children aged 4 to 16 years with indications for adenoidectomy [45]. The primary objective parameter was the A/C ratio measured endoscopically and an obstructive symptom index. After eight weeks, the steroid-treated group showed substantial improvement in the A/C ratio and symptoms, with three-quarters of patients avoiding surgery compared to 80% progressing to surgery in the control group.

Overall, methodological quality appears heterogeneous. Sample sizes were modest [43,44], and observation periods were relatively brief [43]. However, the rigorous triple-blind design of Brouillette’s study lends credibility to its findings [44]. Demirhan’s study supports the potential utility of intranasal steroids in quickly reducing AH [45]. Esteitie’s conclusions are limited, focusing on local inflammatory markers rather than long-term clinical effects [43].

Esteitie et al. reported *p*-values for various inflammatory markers (IL-6, IL-10, etc.), with the lowest, potentially significant value being *p* = 0.05 for IL-6. Despite being marginally significant, this value is chosen as a reference [43]. Brouillette et al. reported severity indices for OSA with *p*-values: AHI (*p* = 0.04), ODI (*p* = 0.03), arousal index (*p* = 0.05), and desaturation frequency (*p* = 0.030). As AHI is typically the primary endpoint in OSA studies, *p* = 0.04 was selected [44].

Demirhan et al. reported *p* < 0.05 for total symptom reduction and A/C ratio improvement without exact values provided. For calculation purposes, a conservative intermediate value of *p* = 0.02 was assumed [45]. A χ^2^ value of approximately 20.25 with 6 degrees of freedom corresponds to *p* ≈ 0.002. Thus, Fisher’s combined analysis indicates a significant overall effect (*p* < 0.01) favouring fluticasone treatment compared to control.

The two studies mentioned in Table 5 [46,47], while sharing the goal of evaluating the effectiveness of intranasal flunisolide in reducing AH and related obstructive symptoms, present differing methodological features.

The study by Ciprandi et al. involved a sample of 178 children aged between 3 and 6 years, divided into a larger group treated with flunisolide (n = 139) and a control group receiving saline solution (n = 39) [46]. The treatment duration was eight weeks, with flunisolide dosage based on body weight, specifically 0.5 drops × kg per nostril, twice daily, administered via an aerosolisation device (Rinowash).

More than 70% of subjects in the treatment arm showed a significant reduction of adenoid tissue, compared to approximately 30% in the placebo group. Many children with grade IV AH improved to lower levels, thus avoiding surgery. However, randomisation appeared imbalanced, and it was unclear whether a genuine blinding procedure was applied [46].

The study by Varricchio et al., for which only the abstract is available, refers to a sample of 178 children with grade III or IV AH. The duration of flunisolide treatment was also eight weeks, with a longer follow-up (at 6 and 12 months post-treatment). Reported data indicate a statistically significant reduction in AH severity by the end of treatment (*p* < 0.01) [47]. The maintenance of benefit was more stable in allergic children (*p* < 0.05).

Each study reported multiple outcomes. We selected *p*_1_ = 0.02 [46] and *p*_2_ = 0.01 [47]. For a χ^2^ distribution with 4 degrees of freedom, a value of 17.03 corresponds to *p* < 0.001. The combination of these *p*-values derived from the two studies indicates a highly significant effect of flunisolide in reducing AH compared to controls [46,47].

Several clinical studies have focused on the treatment of AH and SDB with steroids in combination with other drugs (Table A2).

One study utilised a double-blind, double-dummy design involving 240 children, initially divided into two groups: mometasone furoate vs. placebo. Subsequently, non-responders were assigned to four combination groups with or without oxymetazoline. The results provide evidence of a significant reduction in the A/C ratio and an improvement in symptom scores (snoring, nasal congestion, rhinorrhoea) [48]. The efficacy appears to be enhanced in the presence of the nasal vasoconstrictor.

Another study compared the use of fluticasone spray with a repeated course of azithromycin in 39 children with AH and OSA symptoms [49]. Although a placebo group was lacking, the study design included two randomised active treatments. Overall efficacy was similar between the two treatments, with a slight advantage for the fluticasone-treated group in residual apnoea parameters (*p* = 0.020) [49]. However, the study had a small sample size (n = 39) and an open-label design. Therefore, while the data are encouraging, they should be interpreted cautiously.

Finally, Evangelisti et al. evaluated the effect of combined therapy with oral betamethasone and intranasal beclomethasone for one week, compared to intranasal beclomethasone alone in 28 children with severe OSAS [50]. The results showed significant differences in the improvement of oxygenation (mean and minimum SpO_2_) and acute symptoms, favouring the group receiving systemic steroids [50]. However, the absence of a placebo group reduces the experimental rigour.

The studies listed in Table 6 focus on the use of montelukast in children with AH and/or mild-to-moderate OSAS. These studies predominantly follow a randomised, placebo-controlled methodological design [13,51,52], sometimes with a double-blind approach [13,14,52,53]. The recruited samples are not always large [14,53], and the follow-up duration varies from one month [53] to three to four months [13,14,51,52].

Some research focuses on reducing AH and nasal obstruction symptoms [51,52]. Other studies have conducted polysomnographic assessments: AHI and ODI were decreased significantly in children treated with montelukast [13,14]. However, in Goldbart et al.’s study, the reduction in AHI did not reach full statistical significance (*p* = 0.07) [14], while the OAI and adenoid size showed significant improvement (*p* = 0.01). Conversely, in Kheirandish-Gozal et al.’s study [13], montelukast was associated with a marked reduction in AHI, with *p* < 0.0001, along with benefits in tonsillar and adenoidal size [13].

The double-blind, placebo-controlled study by Wang et al., conducted over four weeks, histopathologically assessed adenoid tissue. The group treated with montelukast showed reduced inflammatory infiltration. The limitations of these studies mainly lie in the small sample sizes (treated patients ranged from n = 23 to n = 30) [14,53] and relatively short follow-up periods (minimum four weeks and maximum three months) [14,52,53].

Naqi et al.’s study included several outcomes (endoscopy, radiography, snoring, mouth breathing, etc.) with *p* ≤ 0.0001 [51]. We select *p* = 0.0001 (endoscopy score or radiography). Goldbart et al.’s study reported *p* < 0.001 for the A/N ratio (the lowest) and *p* < 0.01 for OAI, while AHI, desaturation, and SaO_2_ showed *p* > 0.05 [14]. We choose *p* = 0.001 (A/N ratio). Kheirandish-Gozal et al.’s study reported *p* < 0.0001 for AHI and SpO_2_ nadir, *p* = 0.001 for ODI3%, etc. [13]. We take the most classical endpoint (AHI) with *p* = 0.0001. Shokouhi et al.’s study reported *p* < 0.0001 for total symptoms, mouth breathing, and sleep discomfort and *p* = 0.007 for snoring [52]. We use *p* = 0.0001 (total symptom score). Wang et al.’s study reported *p* = 0.029 (number of germinal centres), *p* = 0.024 (cystic cavities), and *p* = 0.040 (inflammatory infiltration) [53]. We select the lowest value, *p* = 0.024 (cystic cavities). With 10 degrees of freedom (2 × 5 studies), a *χ*^2^ value of 76.55 corresponds to a *p*-value < 0.0001.

The data suggest that, in paediatric settings (AH, OSAS, OME, etc.), the use of montelukast is associated with significant improvements in various parameters compared to placebo or control treatment.

The studies in Table 7 focus on administering montelukast with mometasone furoate, compared with using each drug alone or with a placebo. The studies exhibit some heterogeneity regarding experimental design, sample size, and the evaluation tools [54,55,56,57].

Yang et al.’s study involved 195 children with mild-to-moderate OSAS, who were randomised into three groups: montelukast, mometasone, or a combination of both [54]. The study shows that after 12 weeks, all treated groups experienced a significant reduction in AHI and an improvement in minimum SpO_2_, with the most important benefit observed in the combination therapy group.

In the prospective randomised study by Ras et al., the researchers divided 100 patients into montelukast plus mometasone and mometasone alone [55]. The study did not include a placebo group. After three months of treatment and an additional three-month follow-up, the montelukast–mometasone combination resulted in a more marked reduction in adenoid volume than nasal steroid treatment alone.

The studies by TuhanıOğLu and Erkan and Ras et al., which assessed AH control through symptom scores, reported similar findings [55,57]. The combination of montelukast and mometasone significantly reduced adenoid volume and associated symptoms (snoring, obstruction, mouth breathing) compared to mometasone alone. TuhanıOğLu and Erkan also included groups treated with montelukast alone or receiving no treatment. Their findings indicate that any therapeutic option provides benefits compared to the absence of treatment [56].

Most studies propose a treatment duration of at least 8–12 weeks [54,55,56,57], sometimes extending follow-up to six months [55]. In Jafari et al.’s study, the analysis did not detect a statistically significant difference between the montelukast + mometasone group and the mometasone-alone group [56].

Yang et al.’s study reported *p* < 0.01 (AHI), *p* < 0.05 (Min SaO_2_, A/N ratio, mouth breathing), and *p* < 0.01 (snoring). We select *p* = 0.01 [54]. Ras et al.’s study reported improvements with *p* = 0.001, *p* = 0.008, *p* = 0.019, and up to *p* < 0.001 for the A/N ratio [55]. We take the lowest value, *p* = 0.0001, as a conservative estimate of *p* < 0.001. Jafari et al. showed no significant differences between montelukast + mometasone and Mometasone alone (clinical score: *p* = 0.117; A/N ratio: *p* = 0.161) [56]. We select *p* = 0.117. TuhanıOğLu and Erkan reported *p* < 0.05 for all treatments (montelukast, mometasone, and combination) vs. control [57]. Since they did not provide a precise *p*-value, we adopt *p* = 0.05 as a reasonable estimate. For a *χ*^2^ distribution with 10 degrees of freedom, approximately 42.83 corresponds to a *p*-value far below 0.0001.

From a statistical perspective, the combined results of the five studies strongly suggest that montelukast, mometasone, or their combination have a significant positive effect compared to controls. The presence of one study with a non-significant *p*-value (*p* = 0.117) does not invalidate the evidence provided by the other four, which report *p* < 0.05, some well below 0.001.

The studies reported in Table A3 differ from the previous ones in several aspects, making their inclusion in the study incompatible. Gelardi et al.’s study focuses on saline/iodine aerosol therapy [58]. In contrast, Tracy’s study (1998) evaluates the role of antibiotics in combination with beclometasone (or placebo) for the treatment of OME rather than AH [59].

Hood et al.’s study involves populations with sickle cell anaemia [60], where the primary endpoint is improving cognitive performance rather than reducing adenoidal obstruction or OSAS. Sobhy et al.’s study is dedicated to children who have already undergone adenoidectomy and focuses on the prevention of post-surgical recurrences [33].

The publications by Bilgili et al. lack a proper control group, and their primary focus is on eustachian tube dysfunction associated with adenoids rather than simple AH or OSAS [61]. Wang et al.’s study consists of retrospective or descriptive analyses of steroid use [62].

## 4. Discussion

The results of the study converge in demonstrating that intranasal steroid therapy (mometasone, beclometasone, budesonide, fluticasone, flunisolide), whether as monotherapy or in combination with other drugs, leads to clinical and instrumental improvement in AH and related symptoms compared to the control population. The safety profile of the studies is generally reasonable.

An alternative to steroids for paediatric OSA is oral montelukast. Furthermore, combining montelukast and intranasal corticosteroids offers more significant benefits than treating mild OSA alone. The studies also report mild side effects; however, long-term studies are lacking to verify the safety of these treatments in paediatric patients. Overall, the variety of experimental designs and sample sizes suggests a degree of caution in the clinical interpretation of the results.

Although heterogeneity in study design, population characteristics, and outcome measures limits definitive conclusions, the available evidence suggests that intranasal corticosteroids and montelukast are particularly effective in cases of mild-to-moderate OSA [32]. Similarly, montelukast demonstrated efficacy in studies focusing on mild-to-moderate cases [13,14,51,52,53]. Furthermore, combination therapy with mometasone and montelukast appears particularly beneficial in this subgroup [54,55], although not all trials confirmed superiority over monotherapy [56]. In terms of treatment duration, studies with longer follow-up (≥12 weeks) tend to report more substantial clinical and instrumental improvements with mometasone therapy [63]. Similarly, positive outcomes were documented following 12–16 weeks of montelukast treatment [13,52,54,55]. These trends underscore the potential influence of baseline disease severity and treatment duration on therapeutic outcomes.

**Figure A1:**
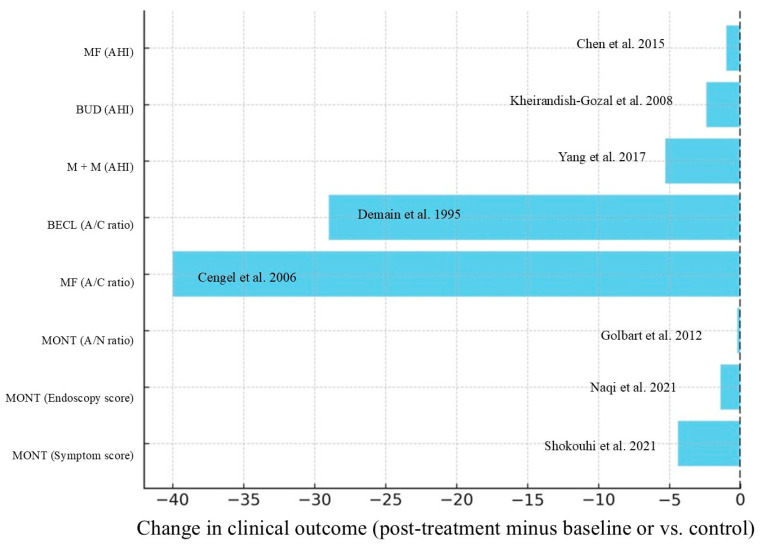
Descriptive forest plot grouped by outcome type [14,32,36,39,42,51,52,54]. Legend: A/C Ratio, Adenoid-to-Choana Ratio; A/N Ratio, Adenoid-to-Nasopharynx Ratio; AHI, Apnoea–Hypopnea Index; BECL, Beclometasone; BUD, budesonide; M+M, combined montelukast and mometasone; MF, mometasone furoate; MONT, montelukast.

It is essential to highlight the variability in clinical outcomes across the different studies. In particular, the reduction in adenoidal volume assessed endoscopically or radiologically, exhibited a variable range. In trials involving mometasone furoate, the A/C ratio decreased from 80% to 40% over six weeks in one study [36], whereas other studies reported more modest or non-significant reductions [34]. Similarly, the improvement in the AHI varied considerably. In patients treated with budesonide, the AHI decreased from 3.7 to 1.3 [42], while in studies involving montelukast, reductions ranged from 9.2 to 4.2 [13] or were not statistically significant (e.g., from 6.0 to 3.6; *p* = 0.07) [14]. In addition, symptom scores also demonstrated considerable heterogeneity. In one study with mometasone furoate, the total symptom score decreased from 11 to 3 [30], whereas in other cases, the reduction was more limited (e.g., from 6.5 to 5.3) [34]. Finally, in some studies, the combination of mometasone and oral montelukast was associated with a more pronounced improvement than monotherapy, showing greater reductions in AHI and endoscopic obstruction [54,55]. However, other studies did not observe significant differences [56]. Although a formal meta-analysis was not performed due to the considerable variability in outcome measures, treatment durations, and study designs, we have extracted and summarised the effect sizes from key studies in Figure A1 shown in Appendix C.

These findings underscore clinical heterogeneity across studies. Therefore, clinical interpretation of results requires caution, and further standardised trials are needed to clarify the actual benefits in different paediatric subpopulations.

Intranasal steroids, treatment duration, and sample size.

The available studies on mometasone furoate have treatment durations ranging from a few weeks [31,36] to one year of follow-up [33]. Typical findings include a significant reduction in endoscopic or radiological obstruction (A/C ratio) [30,36], improvement in polysomnographic and clinical parameters (snoring and apnoea) [30,31,34,36], and a positive effect on patients’ quality of life [35]. Statistical analysis reveals highly significant results.

Studies on beclometasone [37,38,39] are generally double-blind but involve small samples (20–60 children) [38,39]. The treatment duration can extend up to 24 weeks [39]. Two studies did not report statistically significant differences in AH [37,38], while one showed a marked reduction [39]. However, the overall statistical analysis result is substantial.

Similar results emerge from studies on budesonide [40,41,42], with sample sizes ranging from 60 [40] to 100 subjects [41] and follow-up durations of 6 [40,42] to 14 weeks [41]. These studies confirm improvements in respiratory symptoms [40,41] and a reduction in AHI [41].

For fluticasone [43,44,45], a reduction in the A/C ratio [42], a decrease in AHI [44], and an improvement in local inflammation [43] have been observed. The combination of *p*-values highlights a significantly positive overall effect.

For flunisolide [46,47], the results confirm a significant reduction in adenoid volume [46,47]. The available data indicate robust statistical significance (*p* < 0.001).

### 4.1. Intranasal Steroids and Dosages

The available studies show significant variability in the dosages used for intranasal corticosteroids in treating paediatric SDB. Mometasone furoate ranges from 40 µg/day [33] to 200 µg/day [32,34,35], beclometasone from 200 µg/day [37] to 400 µg/day [38], budesonide from 64 µg/day [42] to 1 mg/2 mL nebulised [41], fluticasone from 55 µg/day [43] to 400 µg/day [45], while flunisolide is administered based on body weight or through unspecified dosing regimens [46,47].

This heterogeneity limits the identification of an optimal therapeutic regimen and complicates efficacy comparisons between studies [44], particularly given differences in the study populations (age, severity, comorbidities). Furthermore, chronic use of intranasal steroids raises safety concerns, especially in young children. High-dose treatments require long-term monitoring to identify potential adverse effects [45], such as hypothalamic–pituitary–adrenal axis suppression or reduced growth. Therefore, a personalised therapeutic approach may be appropriate, tailored to the patient’s age [34] and clinical severity, potentially including systemic steroid therapy in more severe cases [50].

Steroids in combination with other drugs (oxymetazoline, azithromycin, betamethasone).

Some studies have explored the use of steroids in combination with other drugs, such as oxymetazoline (mometasone vs. placebo) [48], azithromycin (comparison between fluticasone spray and a macrolide) [49], or oral betamethasone combined with intranasal beclometasone [50]. The addition of a nasal vasoconstrictor (oxymetazoline) or systemic therapy (betamethasone) is associated with an enhancement of the steroid effect.

Statistical analyses from individual studies confirm a significant advantage compared to control groups. However, while these studies provide encouraging preliminary data, methodological limitations—including the absence of placebo groups in two studies and small sample sizes—necessitate caution in interpreting the results.

### 4.2. Montelukast

The studies reviewed on montelukast, both as monotherapy [13,14,51,52,53] and in combination with mometasone [54,55,56], collectively present a positive outlook for treating mild-to-moderate AH and OSAS. Double-blind, placebo-controlled studies [13,51,52,53] offer high methodological evidence. However, one study did not demonstrate improvement across all variables [14]. The effects on specific parameters (e.g., AHI) may vary depending on the clinical presentation’s initial complexity and severity.

The combination of montelukast and mometasone appears particularly beneficial, addressing both inflammatory/leukotriene mechanisms and obstructive components. Studies comparing monotherapy (either montelukast or mometasone) with their combination typically indicate superior outcomes with the combined therapy [54,55,57]. However, one study found no significant differences between mometasone monotherapy and combination treatment [56]. This suggests that synergistic effectiveness may depend on factors such as treatment duration and AH severity. Additionally, a significant proportion of the analysed studies involved small to moderate sample sizes [14,53], potentially limiting statistical power. Furthermore, follow-up durations often did not exceed three to four months [54,55,56,57]. More extended monitoring periods would allow a better assessment of the long-term clinical benefits.

**Figure 2:**
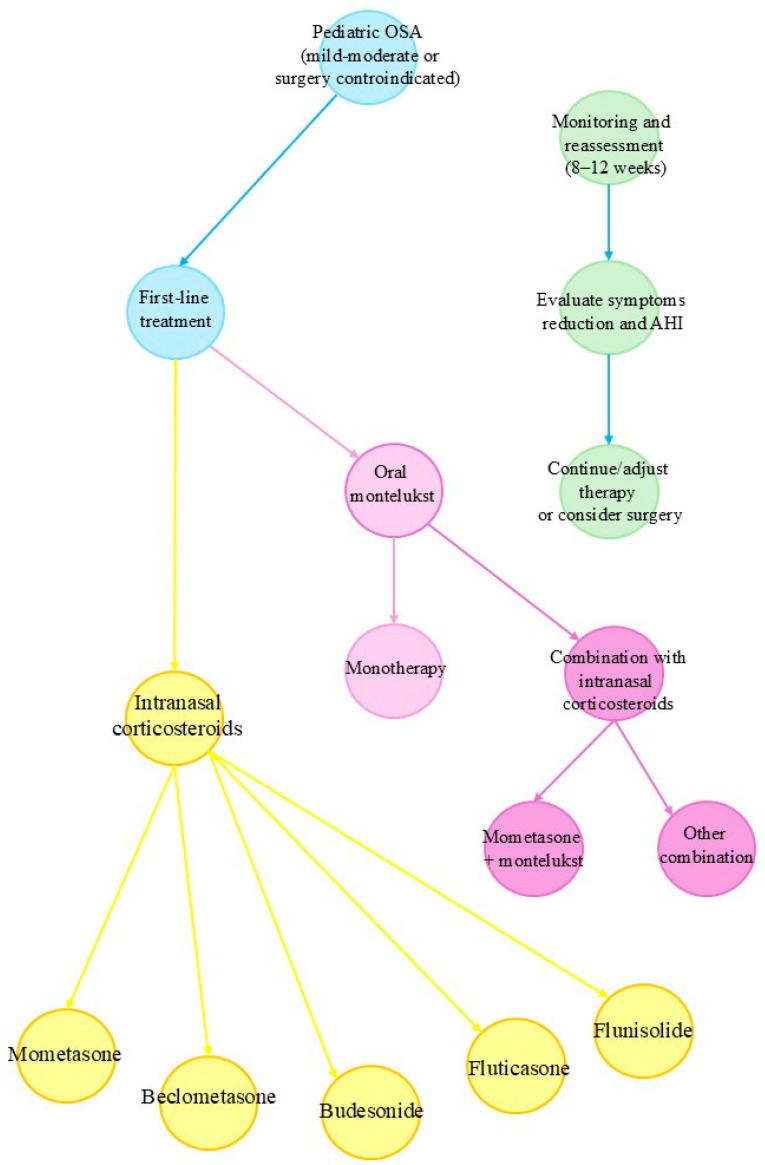
Clinical decision-making flowchart for pharmacologic treatment of paediatric OSA.

Figure 2 shows a flowchart that outlines the pharmacologic treatment approach for paediatric OSA, particularly in cases of mild-to-moderate severity or when surgical intervention is not feasible. It integrates current evidence on using intranasal corticosteroids, oral montelukast, and their combination. The approach emphasises initial evaluation, stratified treatment selection, and reassessment after 8–12 weeks.

Given the reports of neuropsychiatric adverse effects, including agitation, depression, and suicidal thoughts, the U.S. Food and Drug Administration (FDA; 4 March 2020) has issued a boxed warning regarding the use of montelukast, particularly in children. Other regulatory agencies have echoed similar warnings (GOV.UK; 29 April 2024). Therefore, careful consideration is warranted when prescribing montelukast, with a preference for alternative therapies when appropriate. If used, close monitoring of neuropsychiatric symptoms is recommended [64].

### 4.3. Study Limitations

The presence of trials with and without placebo groups and heterogeneous or sometimes absent control groups complicates direct comparisons across studies. Inclusion criteria were not consistently homogeneous. Some studies targeted children with mild OSAS, others included more moderate forms, while others focused on AH associated with diverse clinical scenarios (allergic rhinitis, recurrent otitis media with effusion, etc.). Such variability complicates the interpretation and practical clinical applicability of the findings.

To address this heterogeneity, we employed Fisher’s combined probability test. Although this test does not offer quantitative effect estimates such as risk ratios or mean differences, it indicates the overall statistical significance of the available evidence. However, a more accurate clinical and quantitative evaluation would require meta-analytic analyses based on more homogeneous data, standardised outcomes, measurement units, and known variances. The available literature, however, demonstrates substantial heterogeneity among studies in terms of outcome measures and sample sizes, restricting the possibility of rigorous synthesis.

Another limitation of the current literature is the lack of stratification by OSA severity and treatment duration. Our review highlights that several studies suggest greater efficacy in cases of mild-to-moderate OSA and with longer treatment courses. However, these findings are not consistently reported or statistically stratified, which limits the ability to draw definitive conclusions regarding optimal treatment regimens and clinical indications.

## 5. Conclusions

Findings from this review confirm that intranasal corticosteroid therapy, whether as monotherapy or in combination with other medications, is an effective and safe option for managing AH and mild-to-moderate OSAS symptoms in children. Oral montelukast is validated as an alternative therapeutic option, demonstrating particular benefit when combined with intranasal corticosteroids. Nevertheless, variability in trial designs—including the use or absence of a placebo and the heterogeneity or lack of control groups—complicates direct comparisons across studies. Future prospective studies on larger scales, with longer follow-ups and standardised protocols, are essential to evaluate the effectiveness of different treatment durations and identify patient subgroups most likely to benefit from specific therapeutic strategies. Finally, future studies should adopt consistent criteria to classify OSA severity and evaluate these interventions’ time-dependent efficacy.

## Figures and Tables

**Table 1:** Clinical studies on treating adenoid hypertrophy and sleep-disordered breathing symptoms with mometasone furoate (MF).

Reference	Design	Pop (Age)	N	In/Ex	Tx vs. Ctrl	Dur	O1	O2	Out (Tx)	Out (Ctrl)	Stat
Bhargava (2014) [35]	DB-RCT	Child. 2–12 yrs, AH ± OME	100 (30 vs. 32)	In: AH G3–4 ≥ 3 mo, no resp. prev. treatments Ex: prev. adenoidect., ster. < 1 yr, craniofacial disord., Down synd., recurr. acute inf.	MF 200 µg/day (2 puffs/nostril/day) vs. Saline	24 wks	OME resol., adenoid size reduct.	PTA, sympt., QoL	OME resol. 93%	OME resol. 50%	*p* = 0.04 sympt.; *p* = 0.0001 (adenoid reduct. Tx, NS in Ctrl); *p* = 0.0004 (OME); *p* < 0.0001 (PTA); *p* = 0.0001 (QoL)
Berlucchi (2007) [30]	RCT	Child. 3–7 yrs, AH ≥75%	60 tot (27 vs. 30; 57 compl.)	In: choan. obstruct. ≥75%, 3–7 yrs Ex: tonsil hypert., allergies, ster. < 4 wks, acute inf. < 2 wks, craniofacial disord.	MF 50 µg/day (1 puff/nostril/day) vs. Placebo	40 days + 3 mo maint.	Adenoid pad reduct. (avoid adenoidect.)	Obstr. sympt. (obstruct., rhinorrhea, cough, snoring, apnoea), AE	Choan. obstr. 88.5%→64% Tot sympt. 11→3	76.5%→76% Tot sympt. 10→9	*p* < 0.001 (choan. obstr., total sympt., nas. obstr.), *p* = 0.00005 (snor.), *p* = 0.00034 (apnoea)
Cengel (2006) [36]	CRS	Child. 3–15 yrs, AH ± OME ≥3 mo	122 tot (67 vs. 55)	In: AH/OME ≥ 3 mo, ≥2 ATB courses Ex: ster. < 4 wks, immunodef., MF allergy, craniofacial disord.	MF 100 µg/day (1 puff/nostril/day) vs. No Tx	6 wks	AH reduct. (choan. %), OME resol.	Sympt. improv. (snoring, obstr., mouth breath., apnoea)	OME resol. 42.2%, A/C ratio 80→40	OME resol. 14.5%, A/C ratio 70→80	*p* < 0.001 (OME, A/C ratio, sympt.)
Yilmaz (2013) [34]	DB-RXO	Adolesc. 12–18 yrs, AH	28 (30 init., 2 lost)	In: nasal obstruct. ≥ 6 mo Ex: ster. < 1 yr, immunodef., prev. adenoidect.	MF 200 µg/day (6 wks) vs. Saline, 3-wk wash-out, Tx crossover	6 wks + 6 wks	Adenoid vol. (NS)	Total sympt., QoL	TSS: 6.56→4.31	TSS: 6.50→5.33	*p* = 0.000 (total sympt.), *p* = 0.428 (adenoid vol.)
Baker (2023) [31]	MC-DB-RCT	Child. 3–12 yrs, SDB ≥ 2 wks	276 tot (138 vs. 138) 9.4% lost	In: SDB score ≥ −1, No prev. adenotonsillect., BMI <97th perc., no ster. < 6 wks	MF 100 µg/day (1 puff/nostril/day) vs. Saline	6 wks	SDB sympt. resol.	ENT eval., QoL (PedsQL), PSQ-SDB, OSA-5, parental satisf.	SDB resol. 44%, PSQ-SDB 0.51→0.38, OSA-5 6.2→3.6	SDB resol. 41%, PSQ-SDB 0.53→0.40, OSA-5 5.5→3.8	*p* = 0.51 (SDB), *p* = 0.00 (PSQ-SDB, OSA-5, QoL, satisf.)
Chan (2015) [32]	RCT	Child. 6–18 yrs, mild OSA	62 tot (31 vs. 31; 50 compl.)	In: OAHI 1–5, snoring ≥ 3 nights/wk Ex: craniofacial disord., elev. BMI, nasal/phar. surg.	MF 200 µg/day (2 puff/nostril/day) vs Placebo	4 mo	OAHI change (PSG pre/post)	ODI, snoring freq., adenoid/tonsil size (endosc.), sleep param.	OAHI 2.7→1.7, ODI −0.6, snoring 75%→54.5%	OAHI 2.5→2.9, ODI +0.7, snoring unchanged	*p* = 0.039 (OAHI), *p* = 0.037 (ODI), *p* = 0.031 (snoring)
Sobhy (2013) [33]	RCT	Child. 3–13 yrs post-adenoidect.	200 tot (100 vs. 100)	In: post-adenoidect. with persistent sympt. Ex: ster. < 1 yr, epistaxis, immunodef., genetic/neuromusc. disord.	MF 40 µg/day vs. Saline	12 mo	Sympt. recurrence prev. (nasal obstruct., rhinorrhea, snoring)	QoL, reduct. reintervention	Obstr. 2.31→0.73, Nasal secr. 2.16→0.67, Snor. 2.27→0.79	Obstr. 2.33→1.49, Nasal secr. 2.12→1.53, Snor. 2.25→1.44	*p* = 0.001 (obstr.), *p* = 0.0001 (secr./snor.), *p* = 0.003 (X-ray)

Legend: A/C ratio: Adenoid/Choana ratio; AE: adverse events; compl.: completed; CRS: Controlled Retrospective Study; DB-RCT: Double-Blind Randomised Controlled Trial; DB-RXO: Double-Blind Randomised Crossover; ENT: ear–nose–throat; freq.: frequency; init.: initial; MC: multi-centre; MF: Mometasone Furoate; mo: months; nas.: nasal; NS, not significant; obstr.: obstruction; ODI: Oxygen Desaturation Index; OSA-5: Obstructive Sleep Apnoea-5; PSQ-SDB: Paediatric Sleep Questionnaire; PTA: Pure Tone Audiometry; QoL: quality of life; resol.: resolution; satisf.: satisfaction; SDB: Sleep-disordered breathing; secr.: secretion; snor.: snoring; ster.: steroids; surg.: surgery; sympt.: symptoms; tot: total; wks: weeks; yrs: years.

**Table 2:** Clinical studies on the treatment of adenoid hypertrophy and sleep-related breathing disorder symptoms with beclometasone.

Reference	Design	Pop (Age)	N	In/Ex	Tx vs. Ctrl	Dur	O1	O2	Out (Tx)	Out (Ctrl)	Stat
Lepcha (2002) [37]	DB-RCT	Child. 3–12 yrs	31 tot (5 lost)	In: AH (clinical + X-ray), no ster. < 1 yr, no nasal spray < 2 wks, no immunodef./epistaxis. Ex: see inclusion criteria	Beclom. 200 µg/day vs. Placebo	8 wks	Improv. obstruct. sympt. + adenoid size reduct. (X-ray/endosc.)	Eval. nasal secretion, OME, AE	Nasal Block: −1.54 ± 0.97; Snoring: −1.31 ± 1.18; Endosc.: −0.38 ± 0.51	Nasal Block: −1.69 ± 1.11; Snoring: −1.77 ± 1.01; Endosc.: −0.08 ± 0.28	*p* = NS symptom scores (*p* ≥ 0.07)
Criscuoli (2003) [38]	Rnd single-blind, crossover	Child. ~3.8 yrs	60 tot (53 compl.)	In: obstruct. ≥ 6 mo, AH/NF > 0.5, planned A-T. Ex: ster. < 1 yr, epistaxis, immunodef., URTI < 2 wks	Beclom. 400 µg/day vs Saline (2-wk cross.), then 200 µg/day “open” 24 wks	4 wks (cross.) + 24 wks (open) (total ~28 wks); follow-up 100 wks	≥50% reduct. nasal obstruction index (NOI) score	Freq. adenotonsillect., AE, obstruct. meas. at 24, 52, and 100 wks	Nasal Block: −1.54 ± 0.97; Snoring: −1.31 ± 1.18; Mild improv. X-ray/endosc. (NS)	N/A (placebo 2 wks), then all open-label beclom.	*p* > 0.05 for radiogr. and endosc. diff.; no severe AE reported
Demain (1995) [39]	DB-PC-RXO	Child. 5–11 yrs	20 tot (17 compl. 8 wks, 14 compl. 24 wks)	In: Chron. nasal obstruct. sympt., A/C ratio ~90%, no ster. < 12 mo, no immunodef./epistaxis	Beclom. 336 µg/day (2 puff x2/day) vs. Placebo (8 wks), then “open” 168 µg/day for 16 wks	8-wk cross + 16 wks open (total 24 wks)	Adenoid size reduct. (rhinoscopy: −29% at 24 wks) Obstruct. sympt. reduct. (−82% score)	Reduct. snoring, rhinolalia, enuresis in 8/9, fewer ATB, improv. OME pressures	A/C ratio 91%→−14/−15% at 4 wks, −29% at 24 wks; sympt. score: −82%	Ctrl: minimal ratio changes (0%→−2%); sympt. scores essentially unchanged	*p* = 0.0002 and 0.0006 (A/C reduct.), *p*≈0.05 (sympt.); carryover eff. in RXO

Legend: A/C ratio: Adenoid-to-Choana ratio; AE: adverse events; AH: Adenoid Hypertrophy; ATB: antibiotics; compl.: completed; DB-PC-RXO: Double-Blind Placebo-Controlled Randomised Crossover; DB-RCT: Double-Blind Randomised Controlled Trial; diff.: difference; endosc.: endoscopic; Improv.: improvement; In/Ex: Inclusion/Exclusion criteria; immunodef.: immunodeficiency; mo: months; NF: Nasopharynx; NOI: Nasal Obstruction Index; NS: not significant; OME: Otitis Media with Effusion; obstruct.: obstruction; radiogr.: radiographic; reduct.: reduction; Rnd: Randomised; ster.: steroids; sympt.: symptoms; tot: total; Tx vs. Ctrl: Treatment vs. Control; URTI: Upper Respiratory Tract Infection; wks: weeks; yrs: years.

**Table 3:** Clinical studies on treating adenoid hypertrophy and sleep-related breathing disorder symptoms with budesonide.

Reference	Design	Pop (Age)	N	In/Ex	Tx vs. Ctrl	Dur	O1	O2	Out (Tx)	Out (Ctrl)	Stat
Gudnadottir (2018) [40]	Pros, Rnd, DB, PC	Child. 4–10 yrs with SDB	60 tot (30 vs. 30)	In: SDB ≥ 3 mo, no steroids < 4 wks, no prev. AT surgery, no craniofacial disorders. Ex: acute infections, severe SDB	Budesonide 128 µg/day vs. Placebo	6 wks	OSA-18 reduction (QoL in SDB)	Snoring, apnoeas, nasal obstruction, QoL (VAS), adenoid size	OSA-18: 65.2→45.7 (Δ −19.5)	OSA-18: 54.8→47.3 (Δ −7.5)	OSA-18: *p* = 0.0014 Snoring: *p* = 0.0020 Caregiver concern: *p* = 0.0057 QoL (VAS): *p* < 0.001
Hong (2017) [41]	Rnd, DB	Prepubertal child. 6–8 yrs	100 tot (92 compl.)	In: nasal obstruction + snoring ≥ 6 mo, A/N ratio > 0.5, AHI ≥ 2, scheduled adenoidectomy. Ex: steroids < 6 mo, epistaxis, immunodef., URTI < 2 wks, required tonsillectomy	Nebulised budesonide (1 mg/2 mL/day, 2 wks) + subsequent nasal spray 64 µg/nostril/day vs. Saline 2 mL/day for 2 wks	Tot 26 wks (2 wks DB + 12 wks open-label + follow-up)	NOI reduction ≥ 2 + reduced adenoidectomy rate	Snoring, nasal secretion, OME, AE, growth	NOI: 3.41→1.92 Snoring: 3.39→2.01 Adenoidectomy: 30.77%	NOI: 3.37→3.32 Snoring: 3.42→3.38 Adenoidectomy: 73.33%	NOI, snoring, secretion: *p* < 0.001 Adenoidectomy: *p* = 0.002
Kheirandish-Gozal (2008) [42]	DB, Rnd, crossover	Child. 6–12 yrs with mild OSAS	62 tot (48 vs. 32)	In: mild OSAS (AHI 2–7), habitual snoring, hypertrophic AH Ex: asthma with prev. therapy, recent steroids, immunodef., nasal surg., craniofacial abnormalities	Budesonide 64 µg/day vs. Placebo (saline)	6 wks	AHI and N/P ratio reduction	Sleep architecture, SpO_2_	OAHI: 3.7→1.3 N/P: 0.71→0.57 Nadir SpO_2_: 88.9%→91.4%	OAHI: 2.9→4.0 N/P: 0.77→0.77 Nadir SpO_2_: 90.1%→88.5%	*p* < 0.0001 (AHI, N/P) *p* = 0.004 (SpO_2_)

Legend: AE: Adverse Events; AHI: Apnoea–Hypopnea Index; AT: Adenotonsillar; compl.: completed; DB: Double-Blind; freq.: frequency; immunodef.: immunodeficiency; In/Ex: Inclusion/Exclusion criteria; mo: months; N/P: Nasopharyngeal/Oropharyngeal ratio; NOI: Nasal Obstruction Index; OAHI: Obstructive Apnoea–Hypopnea Index; OME: Otitis Media with Effusion; OSA-18: Obstructive Sleep Apnoea-18 questionnaire; OSAS: Obstructive Sleep Apnoea Syndrome; param.: parameters; PC: Placebo-Controlled; prev.: previous; Pros: Prospective; QoL: Quality of Life; Rnd: Randomised; reduct.: reduction; SDB: Sleep-Disordered Breathing; SpO_2_: Oxygen Saturation; surg.: surgery; tot: total; Tx vs. Ctrl: Treatment vs. Control; URTI: Upper Respiratory Tract Infection; VAS: Visual Analogue Scale; wks: weeks; yrs: years; Δ: delta (change).

**Table 4:** Clinical studies on the treatment of adenoid hypertrophy and sleep breathing disorder symptoms with fluticasone.

Reference	Design	Pop (Age)	n	In/Ex	Tx vs. Ctrl	Dur	O1	O2	Out (Tx)	Out (Ctrl)	Stat
Esteitie (2011) [43]	Rnd, prosp., open-label, parallel-group	Child. 2–12 yrs OSA (AHI ≥5/h)	24 tot (11 vs. 13)	In: OSAS (PSG), scheduled for A-T, BMI < 95th perc., no recent steroids, no uncontrolled asthma Ex: craniofacial anomalies, systemic disorders	Fluticasone furoate 55 µg/nostril/day vs. No Tx	2 wks	Adenoid tissue IL-6 reduction	Other inflamm. markers (IL-10, TGF-β, etc.)	IL-6: 44 (13–311) pg/mL	IL-6: 133 (10–674) pg/mL	*p* = 0.05 (IL-6); NS other cytokines
Brouillette (2001) [44]	Rnd, triple-blind, PC, parallel	Child. 1–10 yrs mild-mod. OSA	25 tot (13 vs. 12)	In: OSA (AHI > 1/h), hypertrophic AH/tonsils, no acute inf., no steroids < 3 wks Ex: severe OSA, craniofacial anomalies, steroid allergies	Nasal Fluticasone: 200 µg/day (1st wk), then 100 µg/day (5 wks) vs. Placebo	6 wks	AHI reduction	ODI, arousal, SpO_2_ desat., adenoid/tonsil size, parental sympt. score	AHI: 10.7→5.8 ODI: 7.0→2.9 Arousal: 6.1→2.7	AHI: 10.9→13.1 ODI: 5.6→5.4 Arousal: 4.1→3.9	AHI: *p* = 0.04 ODI: *p* = 0.03 Arousal: *p* = 0.05
Demirhan (2010) [45]	Prosp., Rnd, PC	Child. 4–16 yrs AH	45 tot (25 vs. 20)	In: AH with indication for A-T, sympt. > 6 mo, no recent steroids, no allergies Ex: allergic rhinitis, turbinate hypertrophy, chronic disorders	Fluticasone Prop. nasal drops 400 µg/day vs Saline	8 wks	Reduction A/C ratio, obstr. sympt., apnoeas, snoring	Total sympt. score, tonsil size, otologic parameters	Total sympt.: 13.72→2.96 A/C: 86.9%→56.2% Surgery avoided ~76%	Total sympt.: 14.85→14.65 A/C: 87.2%→85.2% Surgery 80%	*p* < 0.05 (sympt., A/C) No OR/RR data

Legend: AH: Adenoid Hypertrophy; AHI: Apnoea–Hypopnea Index; A-T: Adenotonsillectomy; BMI: Body Mass Index; desat.: desaturation; IL-6: Interleukin-6; IL-10: Interleukin-10; In/Ex: Inclusion/Exclusion criteria; inf.: infections; inflamm.: inflammatory; mo: months; NS: not significant; obstr.: obstruction; ODI: Oxygen Desaturation Index; OR: Odds Ratio; OSA: Obstructive Sleep Apnoea; OSAS: Obstructive Sleep Apnoea Syndrome; PC: Placebo-Controlled; perc.: percentile; Prop.: Propionate; Prosp.: Prospective; PSG: Polysomnography; Rnd: Randomised; RR: Relative Risk; SpO_2_: Oxygen saturation; sympt.: symptoms; TGF-β: Transforming Growth Factor-beta; tot: total; Tx vs. Ctrl: Treatment vs. Control; wks: weeks; yrs: years.

**Table 5:** Clinical studies on treating adenoid hypertrophy and sleep-related breathing disorder symptoms with flunisolide.

Reference	Design	Pop (Age)	N	In/Ex	Tx vs. Ctrl	Dur	O1	O2	Out (Tx)	Out (Ctrl)	Stat
Ciprandi (2007) [46]	Prosp., parallel-group	Child. 3–6 yrs (mean 4.5)	178 tot (139 vs. 39)	In: AH GIII/IV, surgical indication. No steroids < 1 yr, no acute conditions < 2 wks	Nasal Flunisolide (dose in drops per weight, 2×/day, Rinowash) vs. Saline solution	8 wks	AH grade reduction (I–IV), avoid adenoidectomy	Nasal obstruction sympt., potential surgery prevention, NS AE	AH reduction in 72.6% (AH IV: 41.7%→8.6%; Surgery avoided in 46/58)	AH reduction in 30.7% (AH IV: 15.4%→12.8%)	*p* < 0.02 (overall AH reduction); *p* < 0.04 (GIV reduction)
Varricchio (2009) [47]	[abstract] (Possibly Prosp. study)	Child. with AH GIII/IV (age n.r.)	178 tot (group details n.r.)	In: AH GIII/IV (endoscopic); Exclusions not specified	Nasal Flunisolide (dose n.r.) 8 wks vs. Saline, 12-mo follow-up	8 wks + 6–12 mo follow-up	AH grade reduction (endoscopic)	Maintenance of AH reduction (especially in allergic subjects), need for surgery, NS AE	Significant AH reduction at 8 wks (*p* < 0.01), maintained in allergic subjects (*p* < 0.05)	n.d.	*p* < 0.01 (initial AH reduction), *p* < 0.05 (maintenance in allergic subjects)

Legend: AE: Adverse events; AH: Adenoid Hypertrophy; GIII/IV: Grade III/IV; In/Ex: Inclusion/Exclusion criteria; mo: months; n.d.: Not determined; n.r.: Not reported; NS: not significant; Prosp.: Prospective; Rinowash: Nasal irrigation device; tot: total; Tx vs. Ctrl: Treatment vs. Control; wks: weeks; yrs: years.

**Table 6:** Clinical studies on treating adenoid hypertrophy and sleep-related breathing disorder symptoms with montelukast.

Reference	Design	Pop (Age)	N	In/Ex	Tx vs. Ctrl	Dur	O1	O2	Out (Tx)	Out (Ctrl)	Stat
Naqi (2021) [51]	RCT	Child. 4–12 yrs AH	60 tot (30 vs. 30)	In: Symptomatic AH (snoring, apnoea, mouth breathing) + endoscopic/X-ray confirmation Ex: Obesity, acute infections, prev. A-T, recent ster./ATB	Montelukast 5 mg/day vs. Placebo	3 mo	Adenoid reduction (endoscopy, X-ray)	Improvement in snoring, apnoea, mouth breathing	Endoscopy 3.77→2.37, X-ray 87.23%→51.33%	Endoscopy 3.57→3.43, X-ray 81.16%→77.83%	*p* ≤ 0.0001 (endo, X-ray), *p* = 0.007 (snoring), *p* ≤ 0.0001 (mouth breathing, sleep)
Goldbart (2012) [14]	DB-PC	Child. 2–10 yrs Mild-mod. OSAS	46 tot (23 vs. 23)	In: AHI < 10, habitual snoring, AH Ex: Obesity, craniofacial anomalies, recent ster./mont.	Montelukast 4 or 5 mg/day (12 wks) vs. Placebo	12 wks	AHI reduction, adenoid size reduction	OSAS symptom improvement, saturation	A/N ratio 0.81→0.57, AHI 6.0→3.6, OAI 3.9→1.7	N/A	*p* < 0.001 (A/N ratio), *p* = 0.07 (AHI), *p* < 0.01 (OAI)
Kheirandish-Gozal (2016) [13]	DB-RCT-PC	Child. 2–10 yrs Mild-mod. OSA	64 tot (28 vs. 29; 57 compl.)	In: AHI > 2, no prev. A-T, no recent ster./mont. Ex: Severe OSA, chronic conditions, craniofacial anomalies	Montelukast 4 mg/day <6 yrs, 5 mg/day ≥6 yrs (16 wks) vs. Placebo	16 wks	AHI and OSA severity reduction (PSG)	Improvement in ODI3%, min. SpO_2_, arousal index	AHI 9.2→4.2, ODI3 7.2→2.8, Ad. size 2.4→2.0, SpO_2_ nadir 85.2→91.0	AHI 8.2→8.7, ODI3 7.0→6.8, Ad. size 2.5→2.4, SpO_2_ nadir 84.8→86.1	*p* < 0.0001 (AHI, SpO_2_), *p* = 0.001 (ODI3), *p* < 0.001 (Ad. size), *p* = 0.01 (arousal)
Shokouhi (2015) [52]	DB-RCT-PC	Child. 4–12 yrs AH ≥75%	60 tot (30 vs. 30)	In: AH ≥ 75% (endo), nasal obstr. (snoring, apnoea, mouth breathing) Ex: Prev. A-T, ster./mont. < 4 wks, genetic/systemic disorders	Montelukast 5 mg/day vs. Placebo (12 wks)	12 wks	Adenoid reduction (endo, A/N ratio)	Symptom improvement (snoring, mouth breathing, sleep)	76% adenoid reduction, Sympt. 7.7→3.3	3% adenoid reduction, Sympt. 7.4→6.7	*p* < 0.0001 (total sympt., mouth breathing), *p* < 0.007 (snoring)
Wang (2023) [53]	DB-RCT prosp.	Child. 3–8 yrs Mod-sev. AH	20 tot (10 vs. 10)	In: AH ≥ 50%, no mont. allergy, no acute infections Ex: Tonsillar hypertrophy, OME, sinusitis, recent ster./ATB	Montelukast 5 mg/day vs. Placebo (4 wks)	4 wks	Histopathological evaluation of adenoids (germinal centres, inflammatory infiltration)	Blood lymphocyte count, epithelial cysts, inflammation grade	Germ centres: 8.7, Cysts: 0.0, Inflamm. infiltration: 1.1	Germ centres: 16.5, Cysts: 0.6, Inflamm. infiltration: 2.0	*p* = 0.029 (germ centres), *p* = 0.024 (cysts), *p* = 0.040 (infiltration)

Legend: A-T: Adenotonsillectomy; AH: Adenoid Hypertrophy; AHI: Apnoea–Hypopnea Index; A/N ratio: Adenoid/Nasopharynx ratio; ATB: Antibiotics; compl.: completed; DB: Double-Blind; endo: Endoscopy; germ centre: Germinal Centre; In/Ex: Inclusion/Exclusion criteria; inflam.: Inflammation; mo: months; mont.: montelukast; N/A: not available; OAI: Obstructive Apnoea Index; ODI3: Oxygen Desaturation Index (≥3% desaturation); obstr.: Obstruction; OSA: Obstructive Sleep Apnoea; OSAS: Obstructive Sleep Apnoea Syndrome; PC: Placebo-Controlled; prosp.: Prospective; PSG: Polysomnography; RCT: Randomised Controlled Trial; SpO_2_: Oxygen Saturation; sympt.: Symptoms; tot: total; Tx vs. Ctrl: Treatment vs Control; wks: weeks; yrs: years.

**Table 7:** Clinical studies on treating adenoid hypertrophy and sleep-related breathing disorder symptoms with montelukast and mometasone.

Reference	Design	Pop (Age)	n	In/Ex	Tx vs. Ctrl	Dur	O1	O2	Out (Tx)	Out (Ctrl)	Stat
Yang (2017) [54]	RCT	Child. 2–8 yrs, Mild-mod. OSAS	195 tot (65 Mont., 61 Mom., 57 Comb.)	In: AHI 5–10/h, snoring, obstruct. apnoeas Ex: Prev. A-T, drug allergies, craniofacial anomalies, severe obesity	Montelukast 5 mg/day vs. Nasal Mometasone 50 µg/day vs. Comb. (Mont. + Mom.)	12 wks	AHI reduction, ↑ min SaO_2_	Reduction in symptom scores (snoring, hyperventilation, restless sleep)	AHI: baseline 6.9→1.61, Min SaO_2_: 90.16→94.8, A/N ratio: 0.75→0.50, Snoring: 3.59→1.51 (Data across 3 groups: Mont. baseline 7.25→1.3, Mom. baseline 6.1→1.15)	N/A	*p* < 0.01 (AHI, snoring), *p* < 0.05 (min SaO_2_, mouth br.), *p* < 0.05 (A/N ratio)
Ras (2020) [55]	Prosp. Rnd	Child. 3–10 yrs, AH G3/4	100 tot (50 vs 50)	In: A/N ratio > 50%, AH G3/4, no severe OSAS, no recent ster./mont. Ex: Chronic conditions, craniofacial anomalies, allergies	Nasal Mom. (100 µg/day) + Mont. (4/5 mg/day) vs. Mom. alone	3 mo + 3 mo follow-up	Adenoid volume reduction (A/N ratio), endoscopic improvement	VAS symptom score, recurrence rate	A/N ratio: 52.8 ± 11.3, Endoscopic improvement 68%, Recurrence 23.5%	A/N ratio: 62.88 ± 12.1, Endoscopic improvement 36%, Recurrence 55.5%	*p* = 0.001–0.02 (rhino, mouth br., snore, A/N ratio, endosc., recurrence)
Jafari (2024) [56]	Rnd, DB, PC	Child. 2–14 yrs, AH	96 tot (51 vs. 45)	In: AH diagnosis (clinical + X-ray), obstruct. symptoms Ex: Drug allergies, genetic/neuromusc. conditions, recurrent infections, ster./ATB < 2 wks	Mont. 5 mg/day + Nasal Mom. (50 µg/puff/na/day) vs. Mom. (50 µg/puff/na/day) + placebo	2 mo	Clinical score reduction (snoring, mouth br., nasal voice)	Reduced need for adenoidectomy, QoL	Clinical score 9.1→6.4, A/N ratio 0.80→0.74	Clinical score 8.9→6.6, A/N ratio 0.80→0.75	*p* = 0.117 (clinical score), *p* = 0.161 (A/N ratio); no significant difference between groups
Tuhanioglu and Erkan (2017) [57]	Rnd, prosp.	Child. 4–10 yrs, AH 3–4	120 tot (4 groups of 30)	In: AH ≥ 50%, obstruct. sympt., no immediate adenoidectomy Ex: Acute infections, craniofacial anomalies, ster. <3 wks, allergies	G1 Nasal Mom. (50 µg/day) G2 Mont. (4/5 mg/day) G3 Mom. + Mont. G4 No Tx (Ctrl)	3 mo	Adenoid size reduction (X-ray, endoscopy)	Symptom improvement (scale 0–10)	Airway clearance: Mont. −22.51%, Mom. −21.76%, Comb. −21.79%; Obstr. sympt. −14.77/−17.03/−17.54%	Ctrl: −12.46% (adenoid), −8.8% (obstr. sympt.)	*p* < 0.05 (vs. control); no significant difference among the 3 Tx

Legend: ↑: increased; AH: Adenoid Hypertrophy; AHI: Apnoea–Hypopnea Index; A/N ratio: Adenoid/Nasopharynx ratio; A-T: Adenotonsillectomy; ATB: Antibiotics; compl.: completed; DB: Double-Blind; endo: Endoscopy; germ centre: Germinal Centre; In/Ex: Inclusion/Exclusion criteria; inflam.: Inflammation; mont.: montelukast; Mom.: Mometasone; mo: months; N/A: not available; obstr.: Obstruction; OAI: Obstructive Apnoea Index; ODI3: Oxygen Desaturation Index (≥3% desaturation); OSA: Obstructive Sleep Apnoea; OSAS: Obstructive Sleep Apnoea Syndrome; PC: Placebo-Controlled; prosp.: Prospective; PSG: Polysomnography; RCT: Randomised Controlled Trial; SpO_2_: Oxygen Saturation; sympt.: Symptoms; tot: total; Tx vs. Ctrl: Treatment vs. Control; wks: weeks; yrs: years.

## Data Availability

Data derived from public domain resources.

## Abbreviations

The following abbreviations are used in this manuscript:

AH	Adenoid Hypertrophy
AHI	Apnoea–Hypopnea Index
ATH	Adenotonsillar Hypertrophy
ADI	Oxygen Desaturation Index
A/N ratio	Adenoid-to-Nasopharynx ratio
A/C ratio	Adenoid-to-Choana ratio
oAHI	Obstructive Apnoea–Hypopnea Index
OME	Otitis Media with Effusion
OSA	Obstructive Sleep Apnoea
PSQ	Paediatric Sleep Questionnaire
PSG	Polysomnography
RCS	Randomised Controlled Study
RCT	Randomised Controlled Trial
SDB	Sleep Disordered Breathing

## Appendix A

Customised and database-specific search terms (MEDLINE (PubMed), Scopus, and Web of Science), combining keywords and synonyms.

PUBMED: (“children” OR “child” OR “infants” OR “infant” OR “pediatric” OR “paediatric”) AND (“sleep disordered breathing” OR “sleep-disordered breathing” OR “sleep related breathing disorder” OR “SDB” OR “upper airway resistance syndrome” OR “UARS” OR “obstructive sleep apnea” OR “obstructive sleep apnoea” OR “OSA” OR “obstructive sleep apnea syndrome” OR “OSAS” OR “sleep apnea syndrome” OR “sleep apnoea” OR “adenoid hypertrophy” OR “adenoidal hypertrophy” OR “adenoid enlargement” OR “tonsillar hypertrophy” OR “tonsil hypertrophy” OR “tonsillar enlargement” OR “snoring” OR “snore”) AND (“corticosteroid” OR “corticosteroids” OR “steroid” OR “steroids” OR “intranasal corticosteroid” OR “nasal corticosteroid” OR “mometasone” OR “fluticasone” OR “beclometasone” OR “budesonide” OR “prednisone” OR “prednisolone” OR “montelukast” OR “leukotriene receptor antagonist”) AND (“Randomised, double-blind, placebo-controlled” OR “randomised controlled study” OR “randomised controlled trial” OR “randomised controlled study” OR “randomised controlled trial” OR “RCT” OR “randomised clinical trial” OR “randomised clinical trial”) NOT review.

SCOPUS: TITLE-ABS-KEY((“children” OR “child” OR “infants” OR “infant” OR “pediatric” OR “paediatric”)) AND TITLE-ABS-KEY((“sleep disordered breathing” OR “sleep-disordered breathing” OR “sleep related breathing disorder” OR “SDB” OR “upper airway resistance syndrome” OR “UARS” OR “obstructive sleep apnea” OR “obstructive sleep apnoea” OR “OSA” OR “obstructive sleep apnea syndrome” OR “OSAS” OR “sleep apnea syndrome” OR “sleep apnoea” OR “adenoid hypertrophy” OR “adenoidal hypertrophy” OR “adenoid enlargement” OR “tonsillar hypertrophy” OR “tonsil hypertrophy” OR “tonsillar enlargement” OR “snoring” OR “snore”)) AND TITLE-ABS-KEY((“corticosteroid” OR “corticosteroids” OR “steroid” OR “steroids” OR “intranasal corticosteroid” OR “nasal corticosteroid” OR “mometasone” OR “fluticasone” OR “beclometasone” OR “budesonide” OR “prednisone” OR “prednisolone” OR “montelukast” OR “leukotriene receptor antagonist”)) AND TITLE-ABS-KEY((“Randomised, double-blind, placebo-controlled” OR “randomised controlled study” OR “randomised controlled trial” OR “randomised controlled study” OR “randomised controlled trial” OR “RCT” OR “randomised clinical trial” OR “randomised clinical trial”)) AND NOT (DOCTYPE(review))

WEB-OF-SCIENCE: TS = ((“children” OR “child” OR “infants” OR “infant” OR “pediatric” OR “paediatric”) AND (“sleep disordered breathing” OR “sleep-disordered breathing” OR “sleep related breathing disorder” OR “SDB” OR “upper airway resistance syndrome” OR “UARS” OR “obstructive sleep apnea” OR “obstructive sleep apnoea” OR “OSA” OR “obstructive sleep apnea syndrome” OR “OSAS” OR “sleep apnea syndrome” OR “sleep apnoea” OR “adenoid hypertrophy” OR “adenoidal hypertrophy” OR “adenoid enlargement” OR “tonsillar hypertrophy” OR “tonsil hypertrophy” OR “tonsillar enlargement” OR “snoring” OR “snore”) AND (“corticosteroid” OR “corticosteroids” OR “steroid” OR “steroids” OR “intranasal corticosteroid” OR “nasal corticosteroid” OR “mometasone” OR “fluticasone” OR “beclometasone” OR “budesonide” OR “prednisone” OR “prednisolone” OR “montelukast” OR “leukotriene receptor antagonist”) AND (“Randomised, double-blind, placebo-controlled” OR “randomised controlled study” OR “randomised controlled trial” OR “randomised controlled study” OR “randomised controlled trial” OR “RCT” OR “randomised clinical trial” OR “randomised clinical trial”)) NOT DT = (Review).

## Appendix B

**Table A1:** Procedure (Fisher’s combined probability test) for calculating the combination of probabilities (*p*-values) in Microsoft Excel for Windows.

A	B
*p*-values	LN(*p*-value)
A2: *p*-value1	=LN(A2)
A3: *p*-value2	=LN(A3)
...	...
A11: *p*-value10	=LN(A11)
SUM:	=SUM(B2:B11)
X^2^ = −2 × SUM:	=−2 × B12
combined *p*-value:	=CHISQ.DIST.RT(B13, 2 × COUNT(A2:A11))

Legend: LN, natural logarithm; LN(A2), natural logarithmic conversion; SUM(B2:B11), sum of logarithms; (=−2 × B12), Chi-square statistic calculation; 2 × COUNT(A2:A11), degrees of freedom; CHISQ.DIST.RT(B13, 2 × COUNT(A2:A11)), combined *p*-value calculation.

**Table A2:** Clinical studies on the treatment of adenoid hypertrophy and sleep-related breathing disorder symptoms with steroids in combination with other drugs.

Reference	Design	Pop (Age)	N	In/Ex	Tx vs. Ctrl	Dur	O1	O2	Out (Tx)	Out (Ctrl)	Stat
Liu (2017) [48]	Two-stage, parallel, Rnd, DB, DD	Child. 6–12 yrs AH + AR	240 tot (MF vs. Placebo; Stage 2: 4 groups)	In: AH ≥ 75% rhinoph., sympt. ≥ 12 mo, positive allergy tests Ex: Seasonal rhinitis, craniofacial anomalies, recent ster./ATB	Stage 1 (6 wks): MF 50 µg/nostril/day vs placebo Stage 2 (8 wks, non-responders): MF + Oxymet vs. MF + Placebo vs. Placebo + Oxymet vs. Placebo + Placebo	6 wks + 8 wks	A/C reduction, increased nasal volume	Symptom reduction (congestion, snoring, etc.)	A/C: 87.2→27.3; Nasal vol. 11.2→16.8; TSS: 16.5→4.8	A/C: 85.1→83.4; Nasal vol. 11.1→11.2; TSS: 15.3→14.9	*p* < 0.05 (A/C, nasal vol., TSS, congestion, snoring)
Hashemi Jazi (2011) [49]	Prosp., Rnd, longitudinal	Child. 2–10 yrs AH	39 tot (20 Flutic. vs. 19 Azithro)	In: AH + OSA sympt. (apnoea, snoring, hypernasality) Ex: Craniofacial, neuromusc. anomalies, recurrent infections, ster./ATB <4 wks	Nasal Fluticasone (1 puff/nostril × 2/day ×1 wk, then 1/day × 5 wks) vs Azithromycin (12 mg/kg × 5 days in cycles ×6 wks)	6 wks	AH severity reduction, OSA sympt. reduction	Reduced nasal obstr., snoring, mouth breathing	Obstr. 51–100%: 70%→40%; Snoring: 85%→10%; Mouth Breathing: 60%→10%; Apnoea: 10%→5%	Obstr. 51–100%: 79%→47%; Snoring: 95%→5%; Mouth Breathing: 68%→5%; Apnoea: 15%→0%	Obstr. *p* = 0.004; Snoring/Mouth Breathing *p* < 0.001; Apnoea *p* = 0.02
Evangelisti (2022) [50]	Unblinded, open-label	Child. 3–10 yrs Severe OSAS (AHI > 10)	28 tot (15 vs. 13)	In: Severe OSAS (PSG), awaiting A-T Ex: Chronic cardiopulm., craniofacial, genetic disorders, epilepsy, severe obesity	Oral Betamethasone (0.1 mg/kg/day × 7 days) + Nasal Beclometasone vs. Nasal Beclometasone alone	7 days	Improved SpO_2_ (mean, min.), SCR reduction	ODI reduction, time < 90% SpO_2_	SCR: 12.6→8.3; Mean SpO_2_: 95.3→97.0; Min SpO_2_: 78.8→89.2; ODI: 11.7→3.0; <90% time: 1.75→0.0	SCR: 12.2→12.3; Mean SpO_2_: 95.6→95.5; Min SpO_2_: 82.5→77.8; ODI: 12.3→11.3; <90% time: 1.35→2.0	*p* = 0.0001 (SCR, Mean SpO_2_), *p* = 0.001 (Min SpO_2_), *p* < 0.0001 (ODI, desaturation <90% time)

Legend: A/C: Adenoid/Choana ratio; AHI: Apnoea–Hypopnea Index; AH: Adenoid Hypertrophy; A-T: Adenotonsillectomy; ATB: Antibiotics; Azithro: Azithromycin; DB: Double-Blind; DD: Double Dummy; desatur.: Desaturation; Flutic.: Fluticasone; In/Ex: Inclusion/Exclusion criteria; Mom.: Mometasone; mo: months; Nasal vol.: Nasal Volume; Obstr.: Obstruction; ODI: Oxygen Desaturation Index; OAI: Obstructive Apnoea Index; OSA: Obstructive Sleep Apnoea; OSAS: Obstructive Sleep Apnoea Syndrome; PSG: Polysomnography; prosp.: Prospective; Rnd: Randomised; SCR: Sleep-Related Complaints; SpO_2_: Oxygen Saturation; sympt.: Symptoms; TSS: Total Symptom Score; tot: total; Tx vs. Ctrl: Treatment vs. Control; wks: weeks; yrs: years.

**Table A3:** Clinical studies that were excluded from the review after a comprehensive assessment of the full manuscript.

Reference	Design	Pop (Age)	N	In/Ex	Tx vs. Ctrl	Dur	O1	O2	Out (Tx)	Out (Ctrl)	Stat
Gelardi (2013) [58]	Rnd, blinded, PC study	Child. 4–12 yrs AH/sub-obstr. tonsils	45 tot (27 vs. 18)	In: AH/sub-obstr. tonsils, SDB/OME > 6 mo, no immunosuppr. < 3 mo Ex: Cardiac, bronch. disorders, iodine allergy, TB	Aerosal^®^ (micronized NaCl + iodine) 10 sessions vs. Placebo (no aerosol)	~2 wks (10 sessions) + 3-mo follow-up	≥25% hypertrophy reduction (clinical-endoscopic)	Hearing (≥10 dB), tympanograms, SpO_2_, nasal cytology	≥25% reduction: 44.4%; Hearing +5 dB; Tymp. improvement 29.6%	≥25% reduction: 22.2%; Hearing 0 dB; Tymp. 5.6%	*p* = 0.204 (hypertrophy), *p* = 0.018 (hearing), *p* = 0.064 (tymp.)
Hood (2021) [60]	MC, DB, Rnd, CT	Child. 3–7.99 yrs with SCA + SDB	Expected 200 tot	In: SCA (HbSS/HbSβ0), SDB, no current mont. Ex: Adverse mont. reactions, developmental disorders, participation in other trials	Montelukast 4 mg/day vs. Placebo	12 wks	Primary: ↑ cognitive processing speed (secondary: AH reduction)	Executive function, cerebral perfusion, SDB parameters	Mont. (n = 100): Processing speed +8 pts; A/N ratio 0.81→0.57	Ctrl (n = 100): No change; A/N ratio unchanged	Power 90% for Δ = 8 pts cognitive; α = 0.025; ITT analysis
Tracy (1998) [59]	DB-PC-Rnd	Child. 3–11 yrs Chronic OME	61 tot (20 vs. 19 vs. 20) (3 groups)	In: OME > 3 mo, >3 AOM episodes in 6 mo Ex: Systemic ster. <6 mo, tubes, beclom. allergy, nasal spray < 2 wks	1) ATB (amoxic./sulf.) 2) ATB + nasal beclom. 336 µg/day 3) ATB + nasal placebo	12 wks	Middle ear effusion resolution (tympanometry/otoscopy)	Ear pain, hearing, tympanic mobility	G2 (ATB+bec.): Faster effusion resolution, improved tympanic/otoscopy scores (*p* ≤ 0.05 at 4–8 wks)	G1/G3: Slower improvement, “catch-up” at 12 wks	*p* ≤ 0.01 (right tymp. pressure), *p* ≤ 0.004 (symptoms), *p* ≤ 0.05 (effusion)
Wang (2017) [62]	Cross-sect.	Child. <18 yrs with OME	Data on 1.94 × 10^9^ visits	In: OME diagnosis Ex: Acute otitis media, ATB use, recent surgery	Intranasal steroids (various) vs. No use	Retrosp. 2005–2012	Intranasal steroid prescription frequency in OME	QoC implications, appropriateness	Steroids 10.0% (95% CI 6.3–15.5) OR = 3.58	Steroids 3.5% (95% CI 3.1–3.9)	*p* = 0.002 (OR), *p* < 0.001 (risk diff.)
Sobhy (2013) [33]	Rnd prosp. parallel	Child. 3–13 yrs Post-adenoidectomy	200 tot (100 vs. 100)	In: Adenoidectomy, residual symptoms, no ster. < 1 yr Ex: Epistaxis, immunodef., genetic/neuromusc. disorders	Mom. furoate 40 µg/nostril/day × 12 wks vs. Saline	12 mo	Prevention of nasal obstr., rhinorrhea, post-surg. snoring recurrence	QoL, re-surgery reduction	Nasal obstr.: 2.31→0.73; Discharge: 2.16→0.67; Snoring: 2.27→0.79	Nasal obstr.: 2.33→1.49; Discharge: 2.12→1.53; Snoring: 2.25→1.44	OR = 2.89 *p* = 0.001 (obstr.), OR = 3.21 *p* = 0.0001 (discharge), OR = 2.95 *p* = 0.0001 (snore)
Zhao (2023) [65]	Prosp. Rnd CT	Child. 2–9 yrs AH	93 tot (31 AAT, 32 Xiaoxian + AAT, 30 Montel.)	In: AH + nasal congestion, mouth br., snoring, high OSA-18 Ex: Prev. adenoidectomy, polyps, septal dev., recent ster.	AAT vs. Xiaoxian + AAT vs Mont. 4–5 mg/day	1 mo + 6-mo follow-up	Improvement in congestion, mouth br., snoring	QoL, recurrence rate	OSA-18: 69.56→53.47; Congestion: 1.63→0.50; Mouth br.: 1.56→0.47; Snoring: 1.88→0.38; Sec. sympt.: 3.0→0.72	OSA-18: 67.43→58.50; Congestion: 1.67→0.57; Snoring: 1.87→1.0; Sec. sympt.: 2.87→1.32	OSA-18: *p* = 0.004, Open-mouth *p* = 0.006, Snore *p* = 0.008, Sec. sympt. *p* = 0.0014
Bilgili (2023b) [61]	Prosp. Rnd longit.	Child. 4–14 yrs ET dysfunction + AH	100 tot	In: AH, ETD, snoring, mouth br., no prev. surgery Ex: Acute nas./otol. infections, craniofacial disorders, recent ster./ATB	Azelastine + Flutic. dipr. 2×/day (548 µg + 200 µg total/day)	3 mo, No Ctrl	A/C reduction (endoscopic), ETS-7, tubomanometry	OME resolution, nasal symptoms	A/C 82→37%, ETS-7: 6.36→9.72, OME resolved 66%	N/A (no ctrl)	*p* < 0.01 (A/C), *p* = 0.001 (ETS-7, adenoid volume)
Bilgili (2023a) [66]	Prosp. Rnd longit.	Child. 4–13 yrs AH	65 tot No Ctrl	In: AH > 6 mo, no ster. < 4 wks, no allergies/atopy Ex: Prev. surgery, craniofacial disorders	Azelastine+Flutic. (MP-AzeFlu) 2×/day (548 µg azel. + 200 µg flutic.)	24 wks, No Ctrl	A/C reduction, symptom improvement (obstr., snoring, apnoea, etc.)	Need for surgery, QoL	A/C: 3.57→1.74, Total sympt.: 15.63→2.31, Snoring: 2.82→0.62, Apnoea: 2.52→0.22	N/A (no ctrl)	*p* < 0.01– < 0.001 (A/C, all symptoms)

Legend: ↑: increased; A/C ratio: Adenoid/Choana ratio; AH: Adenoid Hypertrophy; ATB: Antibiotics; Azel.: Azelastine; CI: Confidence Interval; Cross-sect.: Cross-Sectional; CT: Controlled Trial; DB: Double-Blind; Δ: Delta (Change); Endosc.: Endoscopy; ETD: Eustachian Tube Dysfunction; ETS-7: Eustachian Tube Score-7; Flutic.: Fluticasone; Immunsoppr.: Immunosuppression; In/Ex: Inclusion/Exclusion criteria; Longit.: Longitudinal; MC: Multi-Centre; Mom.: Mometasone; Mont.: Montelukast; MP-AzeFlu: Combination of Azelastine + Fluticasone; NaCl: Sodium Chloride (Saline); N/A: not available; NS: not significant; O1: Primary Outcome; O2: Secondary Outcome; OME: Otitis Media with Effusion; OR: Odds Ratio; OSA: Obstructive Sleep Apnoea; OSA-18: Paediatric Sleep Questionnaire for OSA; *p*: *p*-value (statistical significance); PC: Placebo-Controlled; Prosp.: Prospective; QoL: Quality of Life; Rnd: Randomised; RR: Relative Risk; SDB: Sleep-Disordered Breathing; Ster.: Steroids; tot: Total; TSS: Total Symptom Score; Tx vs. Ctrl: Treatment vs. Control; Tymp.: Tympanometry; VAS: Visual Analogue Scale; wks: Weeks; yrs: Years.

## Appendix C

The descriptive forest plot illustrates the effect of intranasal corticosteroids and/or oral montelukast in paediatric patients with OSA or AH. As reported in individual studies, the horizontal bars represent the magnitude and direction of change in selected clinical outcomes (e.g., AHI, A/C, symptom scores). Negative values indicate improvement (i.e., reduction from baseline or compared to control).
